# Designing a multi-epitope vaccine against *Shigella dysenteriae* using immuno-informatics approach

**DOI:** 10.3389/fgene.2024.1361610

**Published:** 2024-05-17

**Authors:** Hurria Qureshi, Amina Basheer, Muhammad Faheem, Muhammad Waqar Arshad, Sunil Kumar Rai, Syed Babar Jamal

**Affiliations:** ^1^ Department of Biological Sciences, National University of Medical Sciences, Rawalpindi, Punjab, Pakistan; ^2^ Department of Biomedical Sciences, School of Medicine and Health Sciences, University of North Dakota, Grand Forks, ND, United States; ^3^ Department of Urology, Feinberg School of Medicine, Northwestern University, Chicago, IL, United States; ^4^ Medical University of the Americas Navis, Charlestown, Saint Kitts and Nevis, West Indies

**Keywords:** multi-epitope, *Shigella dysenteriae*, *in silico* vaccine, molecular docking, membrane proteins, shigellosis, multi-drug resistance, comparative analysis

## Abstract

*Shigella dysenteriae* has been recognized as the second most prevalent pathogen associated with diarrhea that contains blood, contributing to 12.9% of reported cases, and it is additionally responsible for approximately 200,000 deaths each year. Currently, there is no *S. dysenteriae* licensed vaccine. Multidrug resistance in all *Shigella* spp. is a growing concern. Current vaccines, such as O-polysaccharide (OPS) conjugates, are in clinical trials but are ineffective in children but protective in adults. Thus, innovative treatments and vaccines are needed to combat antibiotic resistance. In this study, we used immuno-informatics to design a new multiepitope vaccine and identified *S. dysenteriae* strain SD197’s membrane protein targets using *in-silico* methods. The target protein was prioritized using membrane protein topology analysis to find membrane proteins. B and T-cell epitopes were predicted for vaccine formulation. The epitopes were shortlisted based on an IC50 value <50, antigenicity, allergenicity, and a toxicity analysis. In the final vaccine construct, a total of 8 B-cell epitopes, 12 MHC Class I epitopes, and 7 MHC Class II epitopes were identified for the Lipopolysaccharide export system permease protein LptF. Additionally, 17 MHC Class I epitopes and 14 MHC Class II epitopes were predicted for the Lipoprotein-releasing ABC transporter permease subunit LolE. These epitopes were selected and linked via KK, AAY, and GGGS linkers, respectively. To enhance the immunogenic response, RGD (arginine-glycine-aspartate) adjuvant was incorporated into the final vaccine construct. The refined vaccine structure exhibits a Ramachandran score of 91.5% and demonstrates stable interaction with TLR4. Normal Mode Analysis (NMA) reveals low eigenvalues (3.925996e-07), indicating steady and flexible molecular mobility of docked complexes. Codon optimization was carried out in an effective microbial expression system of the *Escherichia coli* K12 strain using the recombinant plasmid pET-28a (+). Finally, the entire *in-silico* analysis suggests that the suggested vaccine may induce a significant immune response against *S. dysenteriae*, making it a promising option for additional experimental trials.

## 1 Introduction

*Shigella dysenteriae* is recognized as a major contributor to bacillary dysentery or shigellosis, transmitted to humans through the consumption of contaminated water or food. Additionally, person-to-person transmission occurs through the fecal-oral route (Nasser et al., 2022). *S. dysenteriae* is among the four serological groups present in *Shigella* bacteria. It stands as the sole bacterial strain categorized under the clinical group of *Dysenteriae Shigella* species. This designation stems from its unique capacity to generate Shiga toxins (Stxs) (Pakbin et al., 2022). *Shigella* poses a substantial disease burden worldwide, ranking among those bacteria that causes of fatal diarrheal cases globally. The most severely affected demographic consists of children under 5 years old in developing countries (Kumar and Omeershffudin, 2020).

It has been reported that shigellosis results in over 200,000 fatalities globally. As of now, there are no widely accessible vaccines for shigellosis. Nonetheless, several candidates are undergoing evaluation in clinical, yielding essential data and insights. The emergence of antimicrobial-resistant strains (AMR) has heightened concerns regarding *Shigella* (Raso et al., 2023). Approximately half of *Shigella* strains in numerous regions worldwide are now resistant to multiple drugs (Farsiani and Sasan, 2020). The administration of antibiotics for common bacterial infections significantly contributes to diminishing disease prevalence and mortality rates. Nonetheless, inappropriate usage or excessive reliance on antibiotics for treating diarrhea exacerbates the problem of antibiotic resistance (Ranjbar and Farahani, 2019). Bacteria employ various antibiotic-resistance mechanisms, including the utilization of efflux pumps to expel drugs, alterations in membrane permeability, and the expression of enzymes capable of modifying and deactivating molecules (Raso et al., 2023).

According to World Health Organization (WHO) various antibiotics have been used such as ceftriaxone, azithromycin, ciprofloxacin, and pivmecillinam to treat shigellosis. However, there is an efficient increase in the resistance to quinolones and cephalosporin compounds further complicates the situation (Ahamed and Giria, 2021). As for vaccine treatment, different vaccine candidates for *Shigella*, including orally administered live attenuated or inactivated vaccines, as well as subunit vaccines administered via injection, have undergone human trials to assess their safety and immunogenicity. The most advanced vaccine against *Shigella* is O-polysaccharide (OPS) conjugates, while successful in adults and older children, did not provide protection against shigellosis in children under 3 years old, who are the most vulnerable population (Desalegn et al., 2024).

The Ipa proteins, particularly IpaB, IpaC, and IpaD, are key components of the *Shigella* type 3 secretion system (T3SS), this complex is vital in order to internalized into epithelial cell, a beneficial aspect of whole-cell vaccine strategies involves including Ipa proteins alongside other potentially protective proteins. A compelling alternative approach to subunit vaccines is the invasin complex, or Invaplex technology, developed by WRAIR (Turbyfill et al., 2022). The initial version of Invaplex, known as native Invaplex or InvaplexNAT, comprised a complex containing Ipa antigens (IpaB, IpaC, and IpaD) and LPS extracted from *Shigella spp*. Three intranasally administered doses were found to be well tolerated and immunogenic in phase 1 studies conducted in the US. However, in a controlled human infection model study, this formulation failed to provide protection. The extent of added protection achieved by including Ipa proteins in the vaccine are presently uncertain. However, available evidence from animal and human studies indicates that the immune response to IpaB and IpaC is likely to augment protection (MacLennan et al., 2022). The combination of these factors underscores the urgent global public health necessity for developing a vaccine against *Shigella.*

This study aims to identify possible vaccine targets against *S. dysenteriae* by using immuno-informatics methodology to design multiepitope vaccine. The discovery of two stable antigenic membrane proteins from the complete reference genome of *S. dysenteriae* strain SD197 holds significance, especially considering its genomic characteristics*. S. dysenteriae* strain SD197, with its fully sequenced genome consisting of a single circular chromosome spanning 4,369,232 nucleotides and encompassing 4,664 genes, provides a thorough genetic profile of this pathogen (Kumar and Omeershffudin, 2020). Lipopolysaccharide export system permease protein LptF and Lipoprotein-releasing ABC transporter permease subunit LolE are surface localizing proteins, showed significant promise as vaccine candidates. The role of LptF in bacterial cell is that LptF interacts with a transmembrane domain (TMD). Additionally, LptF might function as lateral gates. These gates could potentially aid in the passage of lipopolysaccharides (LPS) across the inner membrane and deposits them onto the Lpt periplasmic bridge, which is a structure involved in further transporting LPS to the outer membrane of the cell (Lundstedt et al., 2020). The role of the LolE subunit within this transporter complex is believed to involve recognizing and transporting lipoproteins from the inner membrane to the periplasm. It participates in the energy-dependent translocation of lipoproteins across the inner membrane, collaborating with other components of the Lol system (Okuda and Tokuda, 2009). Additionally, known for their virulence properties, also present themselves as potential peptide-based vaccine candidates against *S. dysenteriae*. In this study, we employed an immune-informatics strategy to analyze the complete proteome of *S dysenteriae* and identify potential vaccine candidates. This approach was chosen due to its capacity to focus on candidates derived from extracellular and outer membrane proteins, which exhibited efficacy in prior animal studies. Moreover, we mapped T and B cell epitopes for these proteins, utilizing the top-ranking epitopes to formulate a multiepitope vaccine. The predicted B-cell epitopes, MHC class I and MHC class II were merged together by using linkers. A comprehensive characterization of various properties of the vaccine candidates such as antigenicity, allergenicity, and physicochemical properties were conducted. Also, Normal mode analysis was carried out on a vaccine candidate to assess its dynamic behavior and flexibility under diverse conditions. The current research aims to develop a highly effective multi-epitope peptide vaccine against *S. dysenteriae* with the goal of effectively controlling its infection.

## 2 Materials and methods

### 2.1 Genomic and core genome analysis

This study conducted a comprehensive pan-genome analysis, utilizing the complete genome sequences of all 27 strains of *S. dysenteriae*. The reference genome and proteome sequences for these strains were obtained from the National Center for Biotechnology Information (NCBI) and UniProt, respectively (Jamal et al., 2017). The core genome of *S. dysenteriae* was determined using the Efficient Database framework. For comparative genome analyses using the BLAST Score Ratios web tool, the algorithm employed the Basic Local Alignment Search Tool (BLASTp) for proteins, utilizing the standard scoring matrix BLOSUM62 and an E-value cutoff of 1 × 10^−5^. Strain SD197 served as the reference for comparative analysis with other *S. dysenteriae* strains. The core genome of *S. dysenteriae* underwent comparison with the human proteome utilizing BLASTp under default conditions (e-value = 0.0001, bit score ≥100, BLOSUM62 scoring matrix, and identity ≥25%). Only proteins lacking homology with the human proteome database were selected (Qureshi et al., 2021).

### 2.2 Identification of therapeutic vaccine targets and membrane protein analysis

Proteins lacking similarity with human proteins were filtered based on their essentiality using BLASTp in the Database of Essential Genes (DEG) (Riasat et al., 2021). To identify essential proteins, we utilized the standard scoring matrix BLOSUM62, an e-value of 0.001, and an identity threshold of at least 25%. Strain SD197 of *S. dysenteriae* was selected for the identification of potential therapeutic vaccine targets. Protein sequences from the SD197 strain were acquired from UniProt, and a subtractive analysis was conducted using the online database CELLO to exclude 34 membrane proteins (Scott et al., 2005; Qureshi et al., 2021).

### 2.3 Prioritization of vaccine targets

The selected 34 membrane proteins were narrow down on the basis of evaluation of its hydropathicity, physicochemical properties, antigenicity score, allergenicity and membrane topology score, which was carried out using ExPASy ProtParam (https://web.expasy.org/protparam/). Additionally, a membrane topology analysis was performed using TMHMM-2.0 (https://services.healthtech.dtu.dk/services/TMHMM-2.0/) to further assess the candidates. Among the potential targets, the prioritized proteins were found to be the Lipopolysaccharide export system permease protein LptF and the Lipoprotein-releasing ABC transporter permease subunit LolE to be antigenic (Rouzbahani et al., 2022).

### 2.4 B cell epitope prediction

The generation of antibodies through B-cell epitopes plays a crucial role in the development of humoral immunity. To enhance the potential of a multiepitope vaccine, B-cell epitopes for both proteins were predicted using IEDB (http://tools.iedb.org/bcell/) with parameters set by previous studies (Rouzbahani et al., 2022).

### 2.5 T cell epitope prediction

MHC molecules play essential roles in the adaptive immune response and have a significant impact on various host immunological reactions. To predict T cell epitopes for both proteins, the IDEB Tepi Tool database (http://tools.iedb.org/tepitool/) was utilized. This database follows a six-step process that involves selecting classes and alleles, and it provides results based on percentile rank and IC50 values.

For MHC class I epitopes, all peptides with a predicted IC50 value of ≤500 nM and a percentile rank of ≤1.0 were chosen as predicted epitopes. On the other hand, for MHC class II epitopes, peptides with a percentile rank of ≤10.0 and a predicted IC50 value of ≤1,000 nM were selected as predicted epitopes, following the criteria proposed by Paul in 2016 (Paul et al., 2016). The predicted epitopes obtained from this analysis ranged from approximately 9–11 amino acids in length.

### 2.6 Antigenicity, allergenicity and toxicity analysis of peptides

After predicting the peptides for B and T cells, an additional analysis was conducted to assess their antigenicity using Vaxijen v2.0 (http://www.ddg-pharmfac.net/vaxijen/VaxiJen/VaxiJen.html). Only epitopes with significant antigenic potential, as indicated by an antigenicity score ranging from 0.4000 to 2.000 were given priority (Saha et al., 2017). To further refine the selection, the predicted epitopes were evaluated for allergenic and non-allergenic properties using AllerTOP v2.0 (https://www.ddg-pharmfac.net/AllerTOP/). Additionally, toxicity level of the epitopes was estimated using ToxinPred, an online tool on the parameter establised by previous studies. As a result, only epitopes that were prioritized as non-toxic and non-allergenic were chosen for thorough evaluation (Basheer et al., 2023).

### 2.7 Population coverage analysis

An important factor in developing an effective multi peptide vaccine is the presence of particular HLA alleles between various populations and ethnic groups worldwide. Different HLA alleles express themselves differently in different ethnic groups around the world (Jalal et al., 2022). To determine the population coverage of the prioritized epitopes, the IEDB population coverage tool was utilized (http://tools.iedb.org/population/).

### 2.8 Construction of vaccine

For the effective and highly immunogenic design of multi-epitopes vaccines, essential components are required such as epitopes, adjuvant, and linkers. In this construct, the integrin-binding motif RGD (arginine-glycine-aspartate) was chosen as the adjuvant due to its remarkable potency among various molecules (Sarkar et al., 2022). This adjuvant was attached at the N-terminal of the construct, merged to the first residue by the EAAAK linker, which has been shown to effectively induce the immune system. To ensure proper arrangement, B cell epitopes were joined together using the KK linker, while MHC class I epitopes were connected using the GGGS linker, and MHC class II epitopes were linked with the AAY linker. The linkers EAAAK, AAY, and GGGS were employed to join epitopes together (Khamjan et al., 2023). Moreover, EAAAK was utilized for rigidity, reducing interference in the adjuvant-receptor interaction, while GGGS contribute flexibility (Sanches et al., 2021). To enhance the effectiveness of the Multiple epitope vaccine construct, GPGPG and KK linkers were utilized to connect epitopes (Mahmoodi et al., 2023). At the C-terminal, a thrombin cleavage site and a 6X-His tag were introduced. ProtParam was used to evaluate the physicochemical characteristics of the vaccine construct. Additionally, vaccine construct was validated by antigenicity, allergenicity, and toxicity to ensure its safety and immunogenicity.

### 2.9 Secondary structure prediction and structure modelling of vaccine construct

The secondary structure of the vaccine construct was predicted using PSIPRED. To obtain a reliable 3D structure, the construct was subjected to trRosetta (https://yanglab.nankai.edu.cn/trRosetta/), which provides five potential structures in PDB format. These structures were visualized using CHIMERA. The best structure among the five models was selected based on the Ramachandran plot, which evaluates the stereochemistry of the protein backbone. Additionally, the structure was further validated using the ERRAT score, which assesses the overall quality and accuracy of the model (Rouzbahani et al., 2022).

### 2.10 Docking interaction of vaccine construct with human toll like receptor 4

The binding sites of vaccine construct were predicted by castp (http://sts.bioe.uic.edu/castp/index.html?201l) and binding residues were of TLR were predicted by C-port haddock (https://alcazar.science.uu.nl/services/CPORT/) prior to docking of Toll like receptor (PDB id: 2Z65) with vaccine construct. Inflammatory responses caused by gram-negative bacteria primarily involve TLR-4. The TLR4 complex is capable of detecting exceptionally low levels of LPS during bacterial infections. This detection initiates the production of diverse pro-inflammatory substances, such as interleukins (e.g., IL-1, IL-6) and tumor necrosis factor (TNF) which assist in the body’s defense against pathogens. Therefore, TLR-4 was selected as the receptor for the potential epitope-based constructed vaccine (Mazgaeen and Gurung, 2020; Zamyatina and Heine, 2020). Some studies documented that Docking is performed by HADDOCK and UCSF CHIMERA was used to visualize the outcomes. PRODIGY (https://wenmr.science.uu.nl/prodigy/) showed the Gibbs free energy and dissociation constant score of docked complexes (Basheer et al., 2023).

### 2.11 Normal mode analysis of docked complex

Normal mode analysis (NMA) is accomplished on iMODS. By integrating the coordinates of the docked complex, iMODs employs NMA to compute molecular motion and structural flexibility. iMODS shows the results deformability plot; eigenvalue; covariance matrix; elastic network (Rouzbahani et al., 2022).

### 2.12 In-silico cloning

The *Escherichia coli* K12 strain is widely utilized as the prokaryotic expression vector for *in silico* cloning purposes. To optimize the codon usage for this vector, the Java Codon Adaptation Tool (JCAT) (http://www.jcat.de/) is employed. JCAT calculates the Codon Adaptation Index (CAI) score and GC content of the improved sequence, ensuring a better fit for expression in the chosen prokaryotic host. After codon optimization, the improved sequence is then inserted into the recombinant plasmid pET-28a (+) using SnapGene software. SnapGene facilitates visualization and manipulation of DNA sequences, allowing for seamless and efficient virtual cloning procedures (Jalal et al., 2022).

## 3 Results

### 3.1 Target protein identification

Filtering out 34 membrane proteins from CELLO database, an extensive analysis was conducted to evaluate their hydropathicity, physicochemical properties, antigenicity and membrane topology scores. Notably, two proteins, Lipopolysaccharide export system permease protein LptF and Lipoprotein-releasing ABC transporter permease subunit LolE, exhibited remarkable stability. Moreover, both proteins displayed a positive GRAVY (Grand Average of Hydropathy) index, indicating their hydrophobic nature (Table 1).

**TABLE 1:** Properties of Lipopolysaccharide export system permease protein LptF and Lipoprotein releasing ABC transporter permease subunit LolE membrane proteins.

Protein	Amino acid	Instablitiy index	Estimated half-life	Aliphatic index	Grand average of hydropathicity (GRAVY)	Theoretical pI	Total helix outside the membrane	Antigenicity score
Lipopolysaccharide export system permease protein LptF	399	26.27 (stable protein)	30 h (mammalian reticulocytes, *in vitro*)	119.89	0.465	9.09	1,159	0.6861
			>20 h (yeast, *in vivo*)					
			>10 h (*Escherichia coli*, *in vivo*)					
Lipoprotein-releasing ABC transporter permease subunit LolE	360	29.57 (stable protein)	30 h (mammalian reticulocytes, *in vitro*)	117.53	0.386	9.26	1,015	0.4101
			>20 h (yeast, *in vivo*)					
			>10 h (*Escherichia coli*, *in vivo*)					

### 3.2 B cell epitope prediction

The prediction of B-cell epitopes was carried out using Immune Epitope Database (IEDB) (http://tools.iedb.org/bcell/). The predicted B-cell epitopes from both proteins were then subjected to analysis and shortlisting based on their antigenicity, allergenicity, and toxicity. These epitopes ranged in length from 8 to 19 amino acids (mers) (Table 2). There was a total of 6 epitopes for the Lipopolysaccharide export system permease protein LptF and 2 epitopes for the Lipoprotein-releasing ABC transporter permease subunit LolE protein.

**TABLE 2:** Predicted B-cell epitopes of both protein.

Protein	B Cell epitopes	Antigenic score
Lipopolysaccharide export system permease protein LptF	AKANPGMAALAQGQFQQAT	0.442
	IGHQAVALDPNDTD	0.5790
	QMDMRTLWNTDTDRA	0.6926
	VNPRQGRVLS	0.9061
	NGGKGKLDPT	2.024
	DTVPVRRLRASFSR	0.58
Lipoprotein-releasing ABC transporter permease subunit LolE	RGRRRGGM	1.0031
	LSGQLDH	0.8104

### 3.3 T cell epitope prediction

In this study, the T cell epitopes of both proteins were predicted using IDEB tepi tool database (http://tools.iedb.org/tepitool/). The prioritized epitopes are about 9-11-mers in length (Supplementary Table S3A). There were 12 MHC Class I epitopes and 7 MHC Class II epitopes identified for the Lipopolysaccharide export system permease protein LptF. Additionally, 17 MHC Class I epitopes and 14 MHC Class II epitopes were found for the Lipoprotein-releasing ABC transporter permease subunit LolE (Supplementary Table S3B).

### 3.4 Population coverage analysis

**FIGURE 1:**
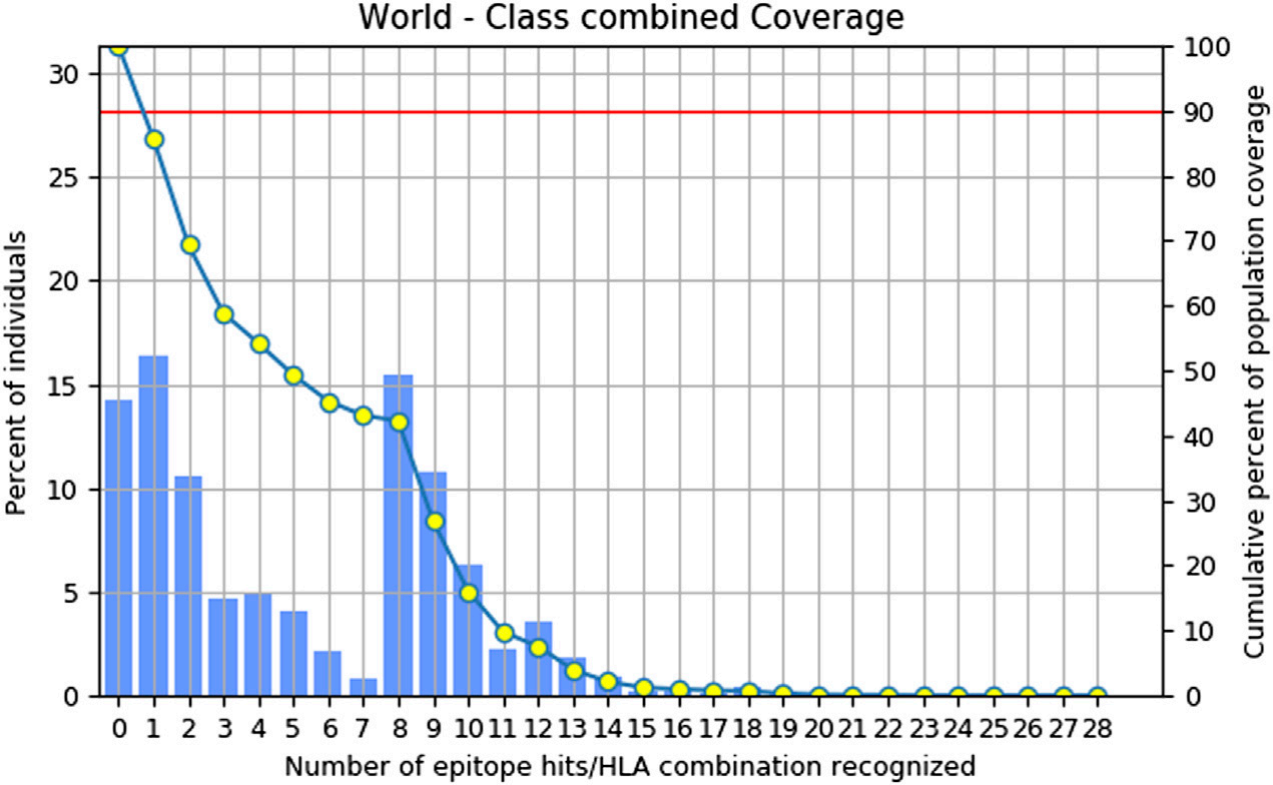
Combinatorial graph of population coverage of prioritized T. cell epitopes.

Combinatorial methods were employed to calculate the population coverage of a specific set of T-cell epitopes, which included epitopes of MHC class I and II T cells, along with their corresponding HLA alleles that bind to them (Sarkar et al., 2022). Approximately, 85.72% of the global population is expected to exhibit a response to the chosen epitopes (Figure 1).

### 3.5 Construction of vaccine

**FIGURE 2:**
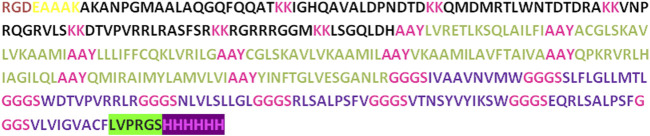
The vaccine construct contains a comprehensive sequence of amino acids. At the N terminus of the construct, an RGD adjuvant has been incorporated. Distinctive linkers (EAAAK, AAY, KK, and GGGS) were introduced to differentiate specific epitopes within the construct.

In the process of developing the vaccine construct, the B-cell and T-cell epitopes predicted earlier underwent further analysis. To mitigate the risk of autoimmune errors, any over-lapping sequences among the leading peptides were carefully removed (Yano et al., 2003). The shortlisted peptides for B-cell epitopes comprised 8 amino acids, while the MHC class I epitopes consisted of 29 amino acids, and MHC class II epitopes were 21 amino acids. Subsequently, the final vaccine construct was assembled by merging all these peptides together and incorporating adjuvants and linkers. At the N-terminal, the adjuvant RGD (arginine-glycine-aspartate), along with GGGS, KK, and AAY linkers, were utilized to connect the peptides. Furthermore, thrombin cleavage and 6-his tags were added at the C-terminal end. As a result, the vaccine construct consisted of 372 amino acid residues in total (Figure 2).

### 3.6 Secondary structure prediction and structure modelling of vaccine construct

**FIGURE 3:**
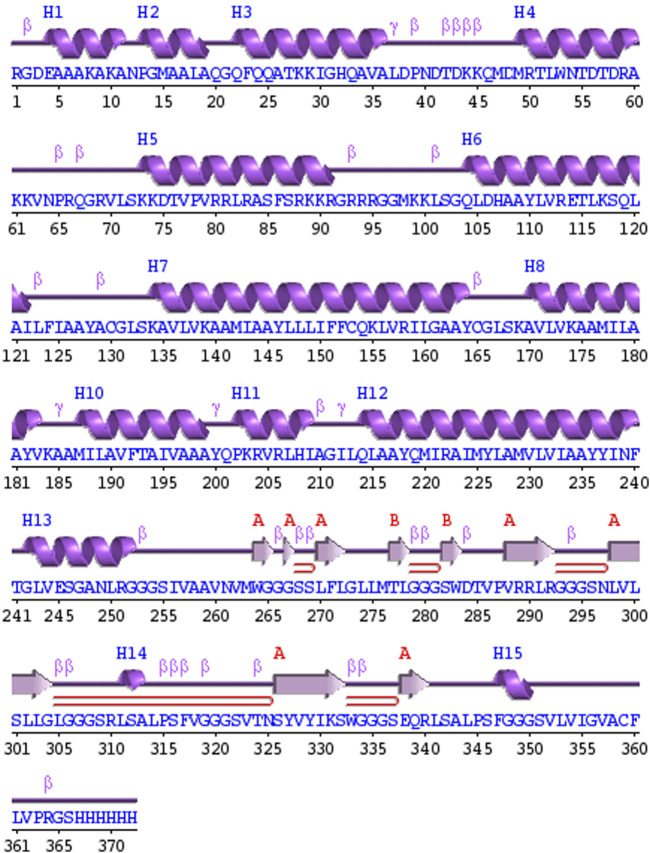
A schematic representation illustrates the secondary structure achieved for the ultimate multiepitope vaccine. The α-helices are denoted as H, while the β-sheets are indicated by labels A and B. The red loops specifically highlight the β-hairpins.

**FIGURE 4:**
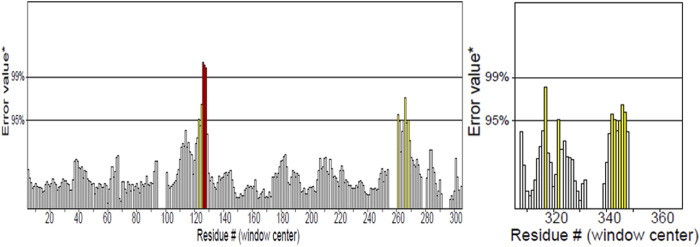
ERRAT showed 95.238 score of vaccine construct, the labeled axis indicating “error” displays two lines that indicate the level of certainty we have in excluding regions where the error values surpass the specified threshold. Residues displaying error values surpassing 99% were identified and marked using the color red. Conversely, areas that exhibit an error value greater than 95% are indicated using the color yellow.

**FIGURE 5:**
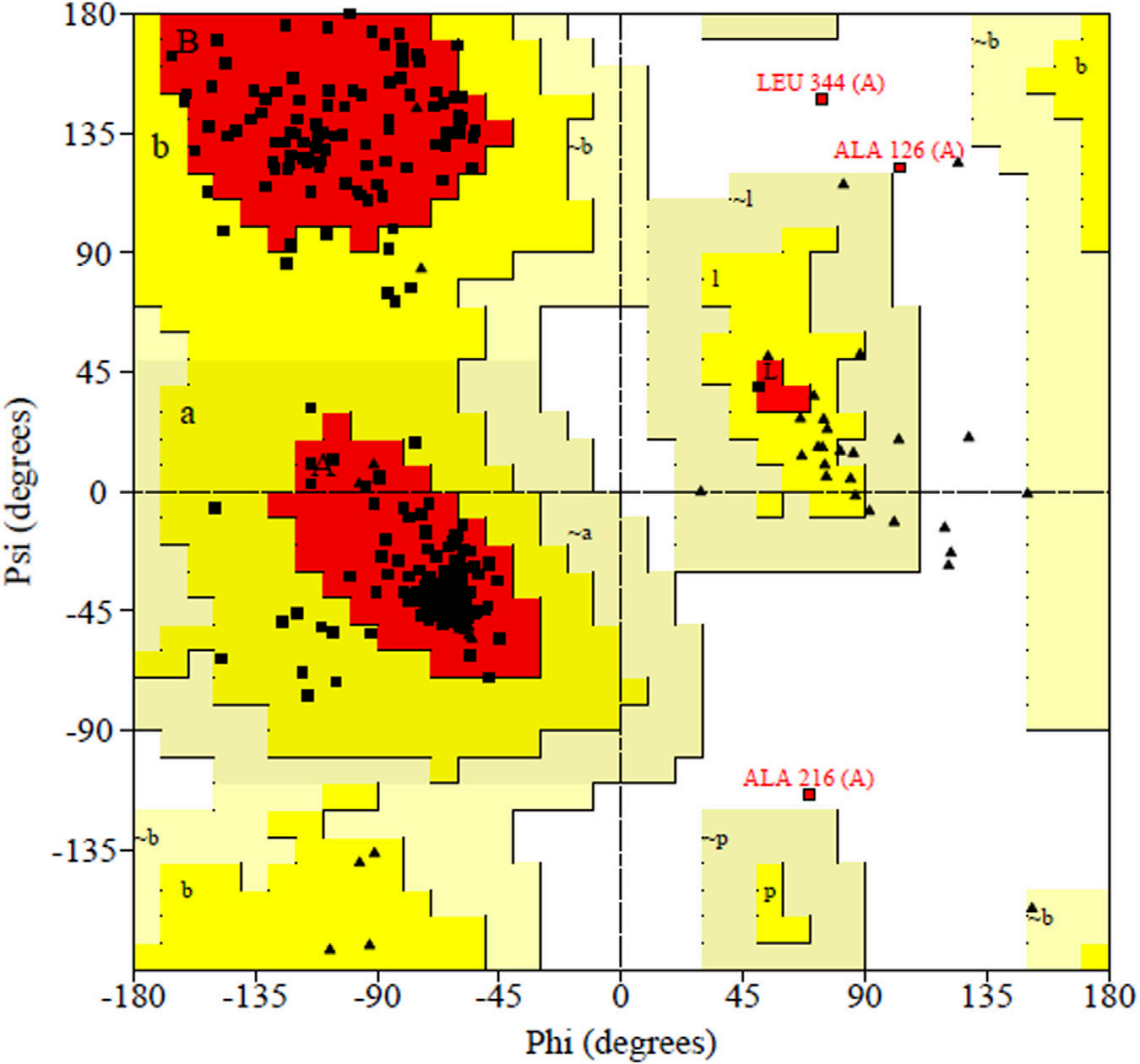
Ramachandran plot shown the most favored regions and additional allowed regions the, the increased red color indicates the residues are present in most favored region.

**FIGURE 6:**
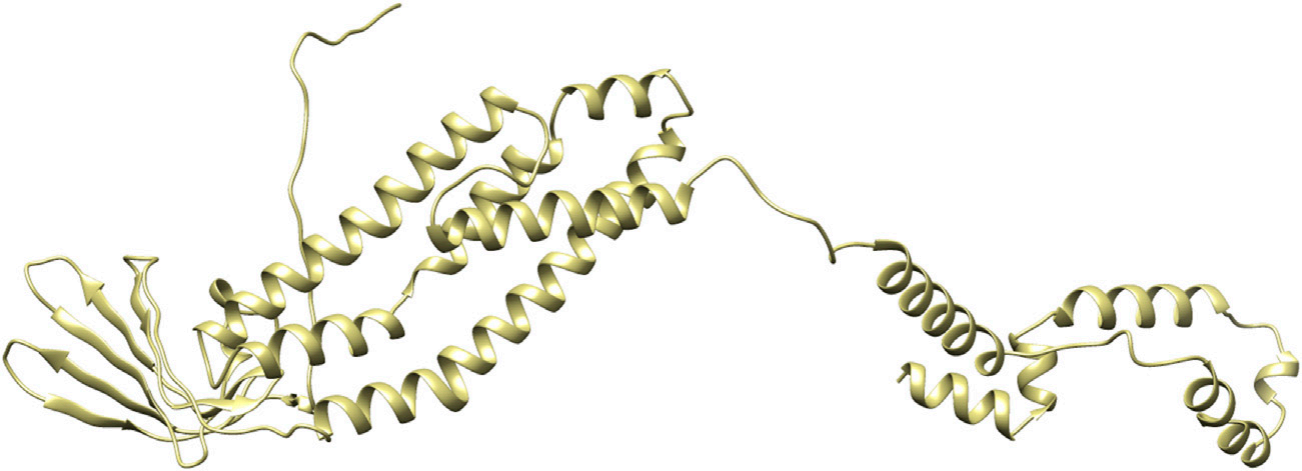
Vaccine construct in 3D visualized on CHIMERA.

PDBsum analysis of vaccine component revealed that it comprises 2 beta sheets, 5 beta hairpin structures, and 15 helices (Figure 3). Meanwhile, trRosetta was utilized to predict the best vaccine construct. The Ramachandran plot demonstrated that approximately 91.5% of residues were in the most favored region, and an additional 7.5% of residues were in the allowed region, indicating good structural quality (Figure 4). The ERRAT score, which was 95.238, further validated the excellent quality of the selected model (Figure 5) (Jalal et al., 2022). Visualization of chosen model was performed using USF CHIMERA (Figure 6).

### 3.7 Docking interaction of vaccine construct with human toll like receptor 4

Before conducting the docking process between the vaccine construct and human TLR-4. For this purpose, Castp database was employed to identify the binding sites for vaccine construct. While the binding site for TLR-4 was predicted on C-port. The database not only provided information about the binding sites but also highlighted the active and passive residues of the TLR-4 protein molecule, which are likely to interact with vaccine construct.

**FIGURE 7:**
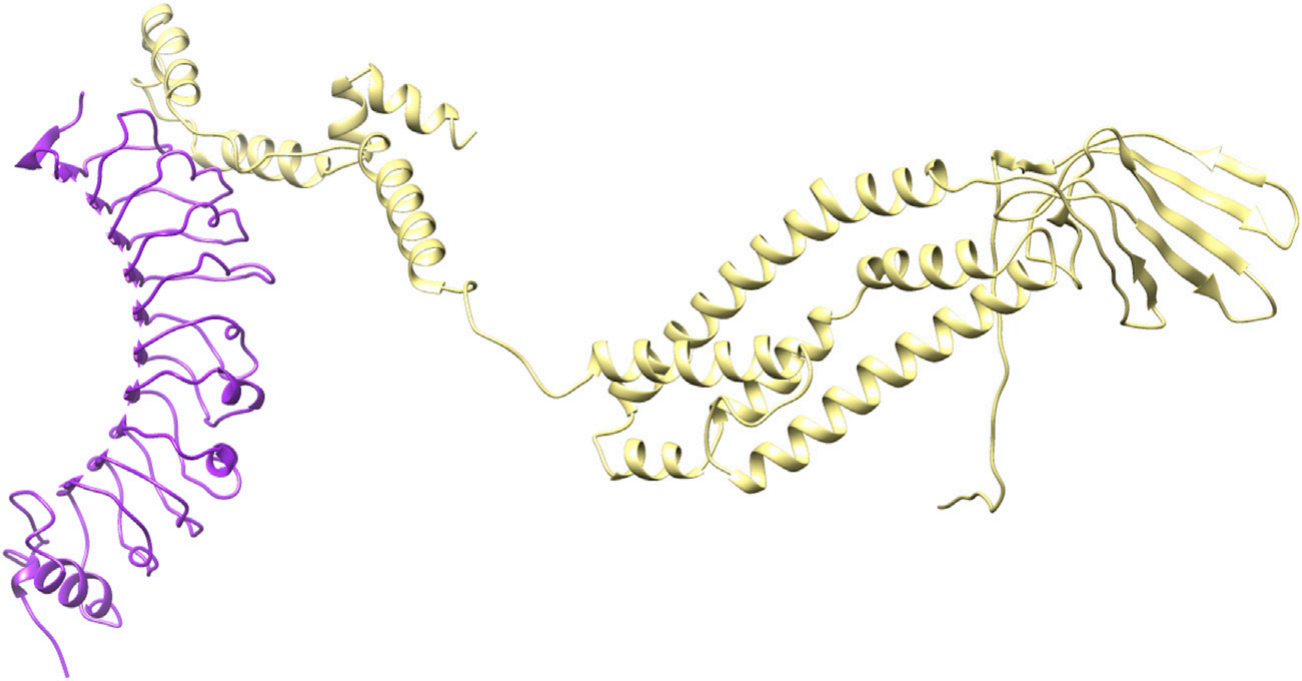
3D structure of docked complex.

**FIGURE 8:**
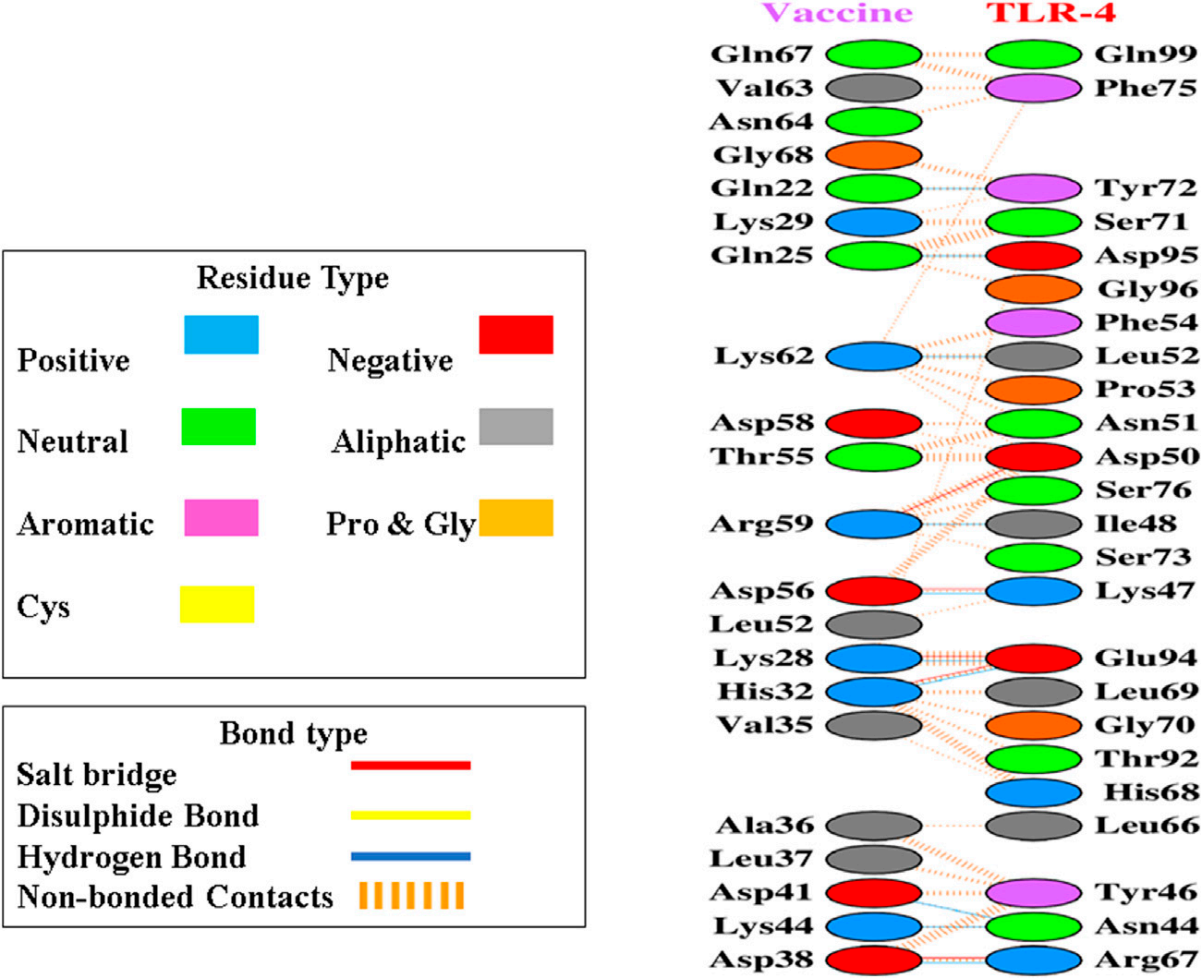
Upon docking, the complex exhibited a considerable number of non-bonded contacts between the vaccine and TLR-4 depicted as dashed lines, along with some noteworthy hydrogen bonds and salt bridges, disulphides bonds as well. Key hydrogen bonds are highlighted in blue, while salt bridges are denoted by red lines. Furthermore, identification of the participating amino acid residues categorized them as aliphatic, aromatic, positive, and negative within these interactions.

After the prediction of active site, the vaccine model was docked with human TLR-4 on online server called HADDOCK. The docking is performed in order to explore the binding affinity, the interactions, and conformation of the docked complex. The docked interactions were visualized on CHIMERA (Figure 7). A negative binding energy provided evidence of a strong and robust affinity between the vaccine and TLR-4 during the interaction process. This negative value indicates a favorable and strong binding affinity between the vaccine and TLR-4. The docked complex was submitted on PDBsum for further analysis, this database revealed that there are 10 binding interactions between the vaccine construct and human TLR-4 (Figure 8). The statistical analysis of HADDOCK docked complex (Table 3). According to PRODIGY predictions, the binding affinity of the docked complex was characterized by a Gibbs free energy (ΔG) of −9.0 kcal mol−1 and dissociation constant (Kd) values of 2.7e-07 M. These results indicate a highly favorable and tight binding between the vaccine and TLR-4 in the complex. The low ΔG value and the small Kd value suggest that the binding between the vaccine and TLR-4 is strong and stable, reinforcing the potential effectiveness of this interaction in the immune response.

**TABLE 3:** The statistical analysis of docked complex.

HADDOCK score	−122.6+/4.0
Cluster size	112
RMSD from the overall lowest-energy structure	28.5+/−0.1
Van der Waals energy (kcal mol−1)	−71.5+/−12.4
Electrostatic energy (kcal mol−1)	−349.7+/−57.4
Desolvation energy (kcal mol−1)	−10.2+/−4.4
Restraints violation energy (kcal mol−1)	290.2+/−76.4
Buried surface area (Å2)	2,171.6+/−25.5
Z-score	−1.5

### 3.8 Normal mode analysis of docked complex

**FIGURE 9:**
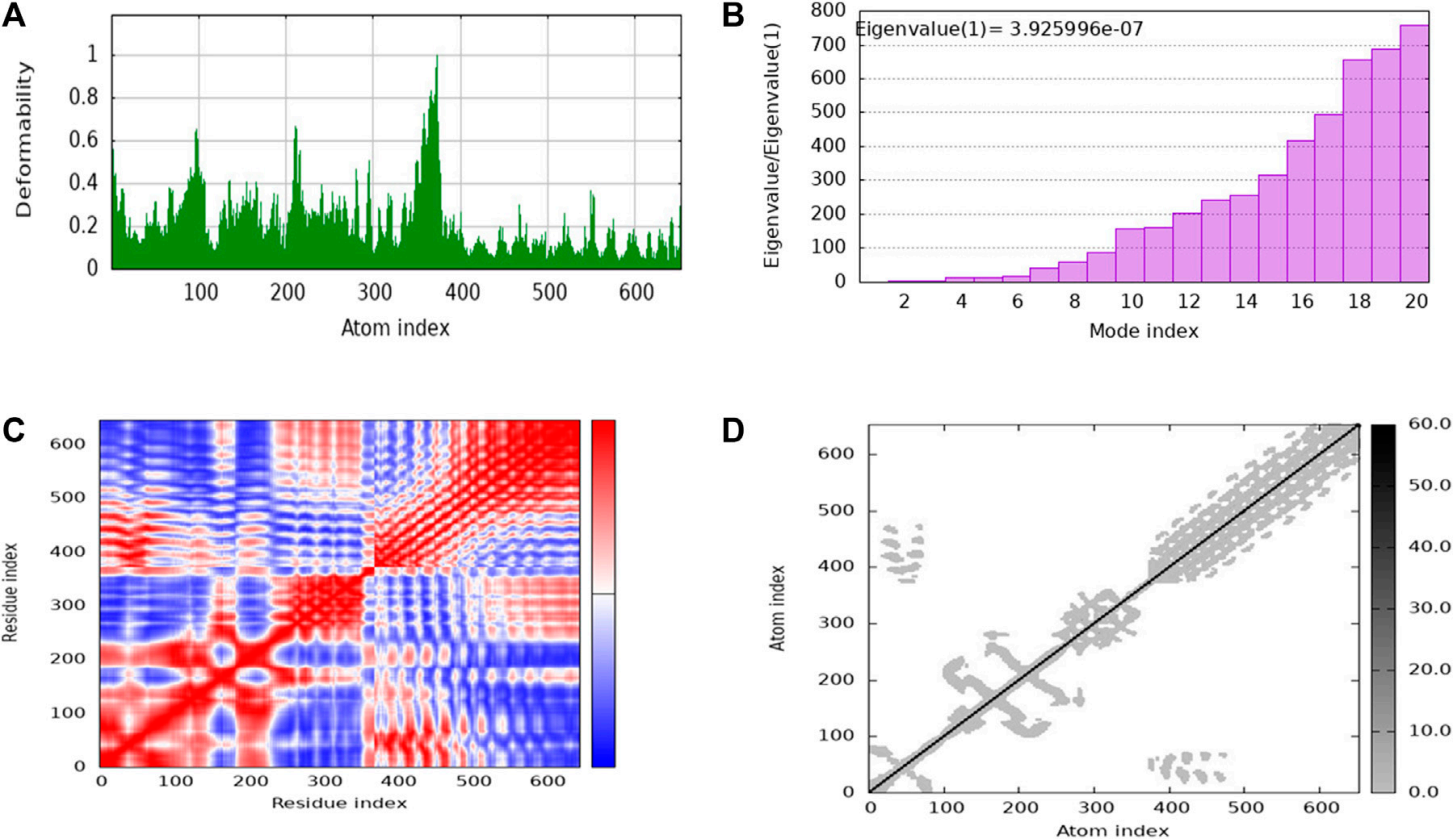
The stability evaluation of the protein-protein complex between the multi-epitope vaccine and TLR-4 utilized molecular dynamics simulation. This analysis encompassed the exploration of **(A)** Deformability, **(B)** Eigenvalue, **(C)** Covariance of residue index and **(D)** elastic network characteristics.

iMODs employs Normal Mode Analysis (NMA) to assess the molecular mobility and structural flexibility of docked complex. The hypothesis behind NMA of proteins is that the lowest frequency vibrational normal modes (referred to as “soft modes”) describes the most significant movements in a protein and are therefore functionally relevant. The study of biomolecular dynamics has been greatly facilitated by normal mode analysis, which is a dependable and effective method. Additionally, it is particularly well-suited for investigating slow dynamics and large conformational changes in protein (Skjaerven et al., 2009; Soltan et al., 2021). The deformability of the complex is primarily determined by the individual distortion of each residue’s Cα atom (alpha-carbon), which is represented by green colored hinges in the chain (Figure 9A). The higher peak indicates higher deformability (Figure 9B) indicated the eigenvalue 3.925996e-07. It relates to the stiffness of the protein structure; lowest eigenvalue indicated easier deformability. The motion between two residues correlates indicated in red color, anti-correlated motions were in blue color, uncorrelated motions were in white color (Figure 9C). In the elastic network model, the connected residues pairs are represented by strings. The dot on the graphs indicates that single spring coinciding to it residues pair, with darker gray indicating a stiffer spring. The stiffness of the springs is represented by the dots (Figure 9D).

### 3.9 Codon optimization and *in silico* cloning

**FIGURE 10:**
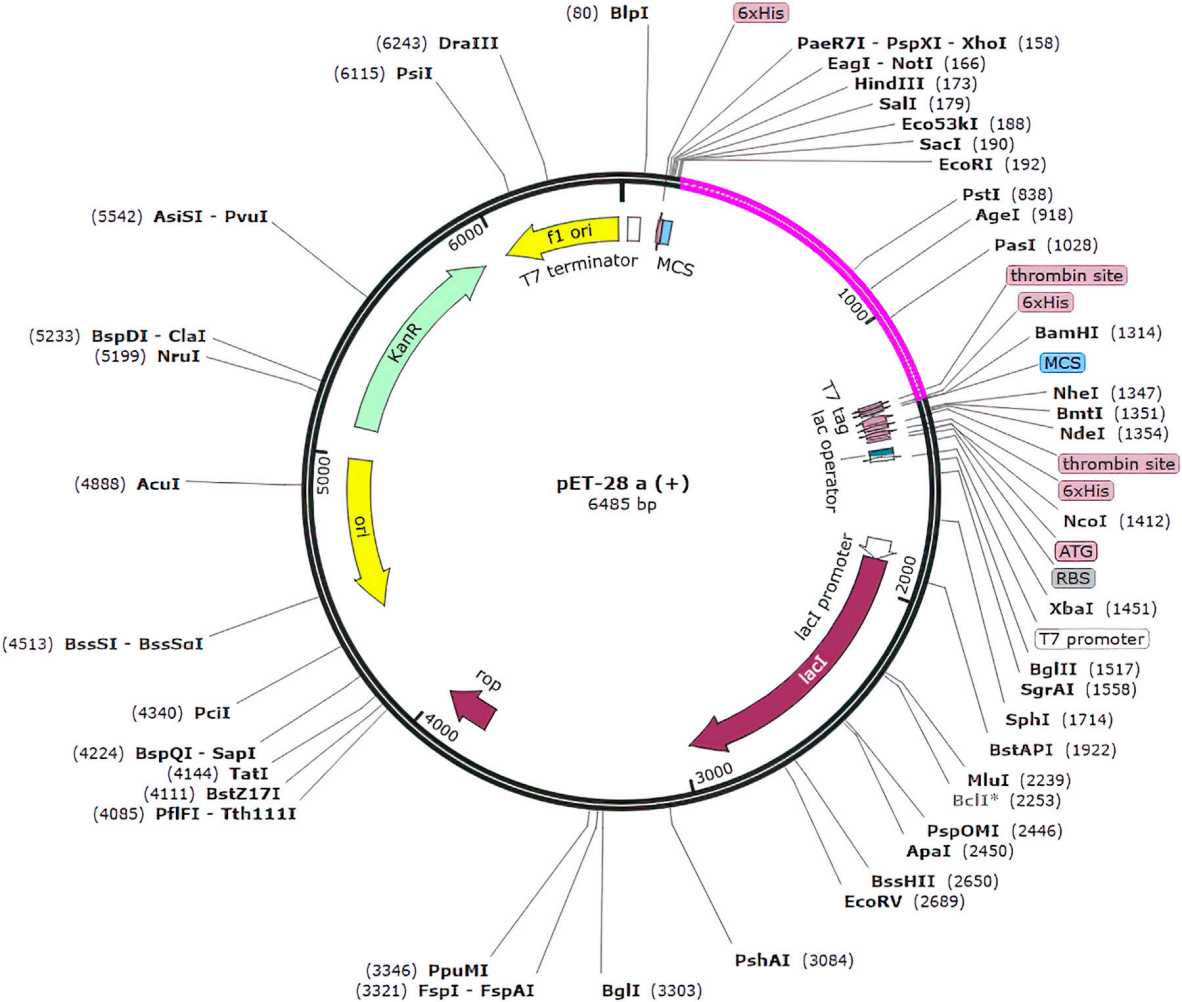
Clone of the designed vaccine construct. The optimized codon sequence of the designed vaccine construct (shown in pink) was cloned between the EcoRI and BamHI enzyme loci in the expression vector pET-28a (+) (shown in black).

In silico cloning was used to determine how our multi-epitope vaccine construct will express on *E. coli* hosts. The vaccine construct was optimized for expression in *E. coli* (K12 strain) using the Java Codon Adaptation (JCat) tool. To mitigate potential errors, we applied two filtering parameters. Firstly, we excluded rho-independent transcription terminators to maintain the integrity of transcriptional processes and prevent premature termination. These terminators are capable of terminating transcription (Łobocka et al., 2004). Secondly, we avoided prokaryotic ribosome binding sites to prevent interference with translation initiation and ensure accurate protein synthesis (Shine and Dalgarno, 1974). These errors can lead to incomplete or inefficient expression of the vaccine construct. The optimized gene had 1,100 nucleotides. The CAI value of optimized vaccine construct was 0.92 (range must be of 0.8–1.0) this value is deemed acceptable as it surpasses the cutoff threshold of 0.8. Furthermore, the G.C. content of the optimized construct was assessed. It was determined that an optimal G.C. content percentage of approximately 30%–70% is preferable for efficient expression in the *E. coli.* The average G.C. percentage of the optimized construct was calculated to be 53.118% (Dey et al., 2022). At end N and C termini of optimized construct, two restricted enzymes (*EcoRI* and *BamHI*) were inserted to facilitate the cloning procedure. We used the SnapGene software to clone the vaccine construct into the pET-28a (+) vector. The size of complete clone was 6.486 kb (Figure 10).

## 4 Discussion

*Shigella* spp. are Gram-negative, rod-shaped bacteria that lack motility and spores. They are responsible for diarrhea, which can advance to bloody mucoid diarrhea, commonly referred to as bacillary dysentery or shigellosis (Kahsay and Muthupandian, 2016). While antibiotic therapy can mitigate the disease, but the rising prevalence of antibiotic resistance in *Shigella* isolates underscores the need for enhanced preventive measures, such as improved hygiene, access to clean water, and the development of pathogen-specific immunity (Walker et al., 2021). Recent studies have shown that *Shigella* have taken the lead of over 200,000 fatalities, which is more common in low-income countries globally (Nuzhat et al., 2022). Even after a century of vaccine development, there remains no licensed vaccine to date for safeguarding against shigellosis, which is the important cause of fatal bacterial diarrhea in children worldwide (MacLennan et al., 2022).

The *in silico* vaccine has gained significant acceptance and due to its duration of development and cost efficiency, it is commonly utilized as the primary stage in vaccine design (Tahir et al., 2019). The *in silico* approach can easily predict whether vaccine construct can induce a strong immune response and overcome major challenges in vaccine development, such as antigenic shifts, genetic dissimilarities. This method offers a valuable tool in creating vaccines with enhanced effectiveness and adaptability to potential viral or bacterial variations (Oyarzún and Kobe, 2016). During the vaccine target identification process, a total of 34 putative membrane proteins were initially identified through the CELLO database. It is widely accepted that a protein’s function is closely tied to its subcellular localization. CELLO has proven to be valuable in predicting the subcellular localizations of proteins identified in proteomic data, aiding in understanding their roles within the cell (Yu et al., 2014). Subsequently, these candidates underwent further filtering based on their antigenicity, allergenicity. Moreover, priority was given to antigens with an antigenicity score ranging from 0.4000 to 2.000. Additionally, their physicochemical properties were evaluated, including amino acid composition, instability index, estimated half-life, aliphatic index, grand average of hydropathicity (GRAVY), theoretical pI, and membrane topology of both proteins. This comprehensive assessment refined the selection, focusing on the most relevant and promising targets.

As a result of this filtering process, two potential protein vaccine targets were identified: Lipoprotein-releasing ABC transporter permease subunit LolE and Lipopolysaccharide export system permease protein LptF. These proteins hold significant potential for the development of multi-epitope vaccine due to their involvement in essential cellular processes and potential immunogenic properties. Epitopes for B and T cells were predicted using the sequences of these potential predicted proteins. Only antigenic, non-allergenic and non-toxic peptides were predicted. The identification of high antigenic epitopes with a Vaxijen score of ≥0.1 guarantees the activation of a robust immune response (Adhikari et al., 2018). There are total 8 peptides of B-cells and 29 peptides of T -cell of both proteins. In a previous study conducted for the development of multi-epitope vaccine by N. Akhtar et al. there were only 3 peptides for B-cell and 5 peptides for T-cells that showed high immunogenicity (Akhtar et al., 2021). *S dysenteriae* displays extensive variability in the human leukocyte antigen (HLA) system, with 95% of the global human population exhibiting diversity in HLA alleles. These include alleles such as HLA-DRB401:01, DRB11501, DRB10401, DRB10701, DRB301:01, DRB10801, DRB11301, DRB10101, DRB10301, DRB11101, HLA-DRB302:02, and HLA-DRB501:01 (Jalal et al., 2022).

After meeting all the required criteria, the peptides were linked together using adjuvants and specific linkers. The adjuvants used in this process were RGD (arginine–glycine–aspartate). The data provided clear evidence that peptides with the RGD motif at the N-terminal side of the -KK- linker significantly induced the production of peptide anti-bodies on the C-terminal side of the -KK- linker (Zamyatina and Heine, 2020), along with GGGS, KK, and AAY linkers, which played a crucial role in facilitating the effective joining of the peptides to form the final multivalent epitope-based vaccine. Through the comprehensive analyses, we designed a vaccine construct that consists of 372 amino acids. The projected vaccine exhibited desirable characteristics, being stable, soluble, highly antigenic, and non-allergenic. These features make it a promising candidate for potential therapeutic applications in combating the target pathogen. Following the design of the vaccine construct, a 3D model was created and its quality was verified by generating a Ramachandran plot. The majority of residues (91.5%) were found within the favored or allowed regions of the plot, indicating that the model exhibited good quality and stability. Furthermore, the ERRAT score for the model was calculated to be 95.238, further confirming the reliability and accuracy of the constructed vaccine model.

Once the 3D model of the vaccine was generated, a molecular docking analysis was conducted to investigate the interactions between the vaccine and the Toll-like receptor molecule 4 (TLR-4). Previous studies have shown the strong activation of TLR-4 and the production of cytokines. The vaccine demonstrated an interaction with TLR-4, exhibiting negative energy in the binding process. This negative binding energy signifies a stronger binding affinity between the vaccine and TLR-4 (Adhikari et al., 2018). It has been documented that TLR4, a receptor found on immune cells, detects lipopolysaccharide (LPS) molecules derived from Gram-negative bacteria. LBP, a protein present in the bloodstream, aids in the transportation of LPS to CD14, which can be found in either soluble or membrane-bound forms on the cell surface. Upon binding to LPS, CD14 transfers it to the TLR4/MD-2 receptor complex, initiating a conformational change in TLR4 and leading to the formation of receptor pairs. This activation triggers intracellular signaling pathways, culminating in the synthesis and release of proinflammatory cytokines such as interleukins (e.g., IL-1 and IL-6) and tumor necrosis factor (TNF), which play crucial roles in the immune response against bacterial infections (Mazgaeen and Gurung, 2020).

The NMA study was conducted to analyze the molecular mobility and comparative deformability of the system. Notably, the calculated eigenvalue of suggests that the docking complex possesses enhanced flexibility. The NMA study was conducted to assess the molecular mobility and comparative deformability of the docked complex. The calculated eigenvalue, 3.925996e-07 indicates that the complex exhibits improved flexibility.

The study verified that the interactions between the vaccine and its targets were balanced. Additionally, *in silico* cloning was successfully carried out, indicating the potential for efficient expression in *E. coli* for large-scale production of the vaccine. Nevertheless, it is essential to validate these findings through *in-vivo* studies. Collectively, these studies suggest that the vaccine candidate holds promise as a prevented therapy against *S. dysenteriae* infections. The significant proteins identified in this study provide a basis for developing a vaccine model aimed at eliciting an immune response against *S. dysenteriae.* Nevertheless, validating the stimulation of a robust immune response against *S. dysenteriae* requires *in vivo* expression and evaluation of the multiepitope vaccine.

## 5 Conclusion

Globally, diarrheal diseases resulting from *Shigella* species are linked to both mortality and long-term dysfunction. This bacterium also tends to get resistant to major antibiotics. Hence, an effective vaccine design is needed cope up with this problem. The *in silico* immuno-informatics approach is demonstrated to develop a multiepitope vaccine which can elicit both innate and adaptive immunity. The vaccine is constructed by predicting the B and T-cells, the antigenicity, allergenicity and toxicity are assessed. The interaction of 3D structure of vaccine construct and TLR-4 showed good binding affinity. Finally, we conducted *in silico* cloning to assess the expression of the final vaccine construct. Our *in silico* approach validates the designed vaccine can stimulate immune response against *S. dysenteriae.* There is a crucial need to carry out *in-vitro* and *in-vivo* studies to confirm its efficacy, immune stimulation.

## Data Availability

The raw data supporting the conclusion of this article will be made available by the authors, without undue reservation.
