# Supplementary material for: Designing a multi-epitope vaccine against Shigella dysenteriae using immuno-informatics approach
Source: Front Genet. 2024 May 17;15:1361610. doi: 10.3389/fgene.2024.1361610 (PMC11143797; doi:10.3389/fgene.2024.1361610)
Supplement: Supplementary file 1 [file Table1.DOCX]

Supplementary Material

Table **S3A**. Predicted MHC class I epitopes of both proteins on the basis of their percentile rank and IC50.

| Proteins | MHC CLASS | ALLELES | PEPTIDES | ANITGENICITY | PERCENTILE RANK | IC 50(≤ 500 nM) |
| --- | --- | --- | --- | --- | --- | --- |
| Lipopolysaccharide export system permease protein LptF | I | HLA-B*58:01 | IVAAVNVMW | 0.4571 | 0.04 | 7.13 |
|  |  | HLA-A*02:03 | SLFLGLLMTL | 0.4593 | 0.17 | 11.19 |
|  |  | HLA-B*57:01 | IVAAVNVMW | 0.4571 | 0.07 | 32.16 |
|  |  | HLA-A*31:01 | RLRASFSR | 0.8517 | 0.24 | 34.13 |
|  |  | HLA-A*02:03 | TLKSQLAIL | 0.5379 | 0.58 | 34.45 |
|  |  | HLA-A*02:06 | SLFLGLLMTL | 0.4593 | 0.4 | 43.77 |
|  |  | HLA-A*68:01 | WDTVPVRRLR | 0.5974 | 0.54 | 58.46 |
|  |  | HLA-B*58:01 | AIVAAVNVMW | 0.4846 | 0.28 | 75.11 |
|  |  | HLA-A*02:03 | NLVLSLLGL | 0.439 | 1.3 | 87.89 |
|  |  | HLA-B*07:02 | RPKGNARPS | 0.5074 | 0.21 | 92.33 |
|  |  | HLA-B*58:01 | LSLFLGLLM | 0.5857 | 0.33 | 93.32 |
|  |  | HLA-A*32:01 | IVAAVNVMW | 0.4571 | 0.12 | 99.96 |
| Lipoprotein-releasing ABC transporter permease subunit LolE | I | HLA-A*02:03 | RLSALPSFV | \| 0.4140 \| \| --- \| | 0.4 | 4.11 |
|  |  | HLA-A*02:03 | YLAMVLVIGV | 0.5873 | 0.09 | 7.46 |
|  |  | HLA-A*02:06 | GQLDHSFAM | 0.5712 | 0.06 | 7.63 |
|  |  | HLA-A*02:01 | YLAMVLVIGV | 0.5873 | 0.09 | 7.46 |
|  |  | HLA-A*02:01 | RLSALPSFV | 0.4140 | 0.12 | 13.18 |
|  |  | HLA-A*02:06 | YLAMVLVIGV | 0.5873 | 0.09 | 7.46 |
|  |  | HLA-B*58:01 | VTNSYVYIKSW | 0.4381 | 0.12 | 24.99 |
|  |  | HLA-A*02:01 | ALKMTDVFNA | 0.5052 | 0.26 | 27.34 |
|  |  | HLA-B*57:01 | VTNSYVYIKSW | 0.4381 | 0.12 | 24.99 |
|  |  | HLA-A*02:03 | ALKMTDVFNA | 0.5052 | 0.67 | 40.5 |
|  |  | HLA-A*30:01 | RVRLHIAGIL | 0.5152 | 0.2 | 41.02 |
|  |  | HLA-B*15:01 | GQLDHSFAM | 0.5712 | 0.16 | 43.93 |
|  |  | HLA-A*02:06 | RLSALPSFV | 0.4140 | 0.46 | 51.35 |
|  |  | HLA-A*02:01 | GQLDHSFAM | 0.5712 | 0.52 | 63.99 |
|  |  | HLA-B*15:01 | KVPGIAAAAPY | 0.5070 | 0.3 | 85.4 |
|  |  | HLA-A*68:02 | YLAMVLVIGV | 0.5873 | 0.48 | 94.25 |
|  |  | HLA-B*15:01 | \| VLVIGVACF \| \| --- \| | 0.5679 | 0.34 | 97.77 |
