# Supplementary material for: Designing a multi-epitope vaccine against Shigella dysenteriae using immuno-informatics approach
Source: Front Genet. 2024 May 17;15:1361610. doi: 10.3389/fgene.2024.1361610 (PMC11143797; doi:10.3389/fgene.2024.1361610)
Supplement: Supplementary file 2 [file Table2.DOCX]

Supplementary Material

Table S**3B**. MHC class II epitopes of both proteins on the basis of their percentile rank and IC50.

| Proteins | MHC CLASS | ALLELES | PEPTIDES | ANITGENICITY | PERCENTILE RANK | IC 50(≤ 1000nM) |
| --- | --- | --- | --- | --- | --- | --- |
| Lipopolysaccharide export system permease protein LptF | II | HLA-DRB4*01:01 | LVRETLKSQLAILFI | 0.4646 | 1.7 | 106 |
|  |  | HLA-DRB1*07:01 | ACGLSKAVLVKAAMI | 0.4737 | 1.9 | 49 |
|  |  | HLA-DRB1*15:01 | LLLIFFCQKLVRILG | 0.4517 | 2.2 | 74 |
|  |  | HLA-DRB1*07:01 | CGLSKAVLVKAAMIL | 0.4005 | 2.4 | 45 |
|  |  | HLA-DRB1*15:01 | EMAQLILPLSLFLGL | 0.5323 | 3.1 | 97 |
|  |  | HLA-DRB1*15:01 | VKAAMILAVFTAIVA | 0.4107 | 5.1 | 98 |
|  |  | HLA-DRB1*07:01 | VKAAMILAVFTAIVA | 0.4107 | 6.6 | 96 |
| Lipoprotein-releasing ABC transporter permease subunit LolE | II | HLA-DRB1*15:01 | DIQMIRAIMYLAMVL | 0.58 | 2.1 | 77 |
|  |  | HLA-DRB1*15:01 | IRAIMYLAMVLVIGV | 0.5165 | 2.1 | 53 |
|  |  | HLA-DRB1*15:01 | QMIRAIMYLAMVLVI | 0.4143 | 2.1 | 50 |
|  |  | HLA-DRB4*01:01 | QPKRVRLHIAGILQL | 0.4347 | 2.5 | 76 |
|  |  | HLA-DRB4*01:01 | QMIRAIMYLAMVLVI | 0.4143 | 4.7 | 95 |
|  |  | HLA-DRB1*07:01 | ISVISTIGIALGVAV | 0.5333 | 4.9 | 67 |
|  |  | HLA-DRB4*01:01 | GVIIGVVVSLQLTPI | 0.5739 | 6.4 | 92 |
|  |  | HLA-DRB1*07:01 | IIGVVVSLQLTPIIE | 0.5077 | 8.4 | 98 |
|  |  | HLA-DRB1*07:01 | GVIIGVVVSLQLTPI | 0.5739 | 8.6 | 33 |
|  |  | HLA-DRB1*07:01 | IRAIMYLAMVLVIGV | 0.5165 | 11 | 76 |
|  |  | HLA-DRB1*07:01 | QMIRAIMYLAMVLVI | 0.4143 | 11 | 96 |
|  |  | HLA-DRB4*01:01 | IRAIMYLAMVLVIGV | 0.5165 | 12 | 87 |
|  |  | HLA-DRB1*07:01 | VISTIGIALGVAVLI | 0.5737 | 12 | 96 |
|  |  | HLA-DRB5*01:01 | YINFTGLVESGANLR | 0.5473 | 17 | 74 |
